# Discussions of Central States Dental Association

**Published:** 1866-06

**Authors:** Geo. W. Field


### DISCUSSIONS OF CENTRAL STATES DENTAL ASSOCIATION.

*Semi-Annual Session, Nashville, Tuesday, April* 10, 1866.

Reported by Geo. W. Field.

### First Day.

The subject of Dental Education was then taken up and discussed. Dr. Taft made some general remarks upon the subject, and then dwelt somewhat at length upon dental colleges, as a means of professional education. He presumed that notwithstanding the existing schools are not all that could be desired, yet they have done much in the right direction for professional education.

They have placed those who have availed themselves of their advantages, upon a basis that they could not otherwise have obtained, both in regard to attainments and position in the profession. He suggested that our colleges have not received the support from the profession that they should have received, and from this fact arises a large portion of the inefficiency that has been attributed to them.

If any one says they are not efficient, and do not accomlish as much as they should, to such an one we reply, put forth your effort to make them better; have you done all you could to make them what they ought to be? He referred to the fact, that some effort has been made to attach dental professorships to medical schools. We will not enter upon a consideration of the merits of this subject, but will say that as it has been advocated in some cases, it will be totally inefficient for good to our profession. He referred to an effort being made to establish a dental school, in connection with the medical department. This enterprise, if established upon the basis proposed, will be successful, as a means of imparting true professional education. He believes that the wants of the profession call for a dental college in the North-West. New York and Boston also should have their dental colleges.

Dr. Shadoan—Understands the establishment of the dental chair in the medical college, as only intended to prepare medical students for a limited country practice of dentistry.

Dr. Cobb—Work, alone elevates. Let us each put forth every effort, and at all times do our best; always laboring to improve ourselves and others. If we find ourselves wanting the qualification to fill well the position we have taken, then withdraw from it, and not remain to be only a drag on the profession, thereby making room for those competent. If, otherwise, then let our light shine, that others may see to follow in the same path. In this manner the inevitable result is to elevate the profession. I will take this opportunity to thank the writer of an article, entitled "Stuffing teeth," in March number of *Register*. Of such operations we have too many, they are no credit, only tending to degrade the profession.

On motion, the next subject was taken up. "Anæsthetics, their proper use and relative value."

Dr. J. L. Nourse—Rarely makes use of any anæsthetic agent, and believed, if systematically opposed, they would soon become obsolete.

Dr. Cobb—had used the battery with moderate success; but had long since discarded it.

Dr. Russell—First commenced the use of chloroform in 1846, have since used it nearly every day, and never have met with an alarming case. I have administered it to patients who had been refused by other operators, and met with universal success. Never have used the nitrous oxide.

Drs. Fouche, Walsh, Hartman, Bedford, Ross, and Redman—had abandoned the use of anæsthetics, believing them to be highly injurious.

Dr. Naghle—I have used the oxide, and was much pleased with its effects. Having administered, and assisted, in at least 4000 cases. I am fully satisfied of the frequent deleterious results, and many times fatal consequences, in the use of chloroform.

Dr. Morgan—Was much opposed to the use of chloroform, owing to the prevailing ignorance, and on this account felt it to be an empirical practice. His fears were first aroused by the fatal accident, from its use, in the hands of Dr. Simpson, the originator.

Dr. Taft—I feel somewhat in the dark on this subject. Many can use chloroform without fear, and with complete success; others with great fear and timidity. He gave several cases of its fatal results, which had come under his immediate observation—all of them in the hands of eminent physicians and surgeons. It does not satisfy me to say that these men know nothing of its nature; we need more light. I do not doubt Dr. Russell's success, yet feel very cautious when I know that so many deaths occur. I cannot explain this difference, unless that some possess an intuitive knowledge, by which they are governed, in forming a proper diagnosis. Do not consider nitrous oxide so objectionable; its action being the reverse of chloroform—I regard it as safe. Do not know of a well authenticated case of death from its use. Its after effects are not so disagreeable; it does not produce the depression and lassitude which follow the inhalation of chloroform. I deem it impracticable for general office use, for the following reasons: The gas must be manufactured *pure*, by a thorough chemist. It may be easily rendered impure in generating, thereby rendering it poisonous. The paraphanalia, for its inhalation, is objectionable. One person should make this a specialty. I would not recommend the use of any anæsthetic—all objectionable—and will never administer chloroform to patient without a physician, believing that it is sufficient for one person to watch its effects, without his attention being absorbed in the operation. The battery, no doubt, would answer in many cases, but owing to the many and varied idiosyncrasies met with, which cannot be fully understood, this, like all other methods, is objectionable.

Dr. Garkey—Says that in Vienna, Austria, there is a chloroform and ether *administerer*, who makes this his whole business. He gives it from a bag, claiming that there was sufficient air mixed with the ether in the bag.

Next subject, "Extraction of teeth; when to extract, and when not."

The discussion was introduced by Dr. Nourse, followed by Dr. Wilson. The former extracted only when no other course would answer. Dr. W., in extracting for artificial denture, always leaves as many as will be required for the benefit of the patient. Would always remove the cuspids, if only they remain, as an artificial denture can be of little use in such a case.

On motion of Dr. Redman, the meeting adjourned to Wednesday, 9 a. m.

WEDNESDAY, 9 O'CLOCK, A. M.

Minutes read and approved. Extraction of teeth resumed.

Dr. Russell—In crowded teeth I extract the bicuspids, and sometimes think that it is wrong. I regard it of much importance, and would like more light on the subject.

Dr. Shadoan—In the extraction of teeth I am governed by two conditions. First, in deciduous teeth, when malformation has or is liable to take place. Second, in adult cases, when resulting from mechanical injury, disease of the alveolus exists. In these cases I always extract. Decayed teeth for children I always prefer to treat and fill. If the patient is not able to pay full amount of bill, will charge only for material used. This course gives us the benefit of experimenting, as well also as the consciousness of having discharged a duty. If there is continued ulceration, would remove its cause.

Dr. Taft—I believe there are too many teeth extracted, yet uniformity of practice can not be obtained among all practitioners, owing to the great difference of ability; some may succeed in the most difficult cases, others fail in accomplishing a desired object, while still others only make a bad job worse. I know that many save all cases presented, no condition of organ being a bar to success, and all should strive to this highest attainment. Now, we all have a slight egotistical opinion of our ability, yet I pity those who can make no further advancement. We should all possess the ability to diagnose correctly each case presented for treatment. Many will not appreciate the efforts necessary to the preservation of a badly diseased organ, while others would not hear to its loss for any consideration. This is another modifying circumstance, and not the mere matter of dollars and cents. We cannot always act benevolently, yet I would never turn off an appreciative, poor patient, when there was time to spare; if such is the case, I always recommend to another operator who will do the patient justice. I hope to see the day when operators will refuse to extract a tooth that can possibly be saved.

Dr. Cobb—Was satisfied that he had made great improvement in this branch, and hoped in the future to devote more time to its study. The education of parents, as to the importance of care for children's teeth, would greatly assist the operator.

Dr. Morgan—I have been many times mortified to see cases that came into my office, they could well be branded as malpractice, Great stress should be laid on the filling of deciduous teeth, owing to the progress of the formative process; then is the time that our attention should be more specially directed to those teeth, in order that a perfectly formed set may be the result.

Dr. Taft—I appreciate Dr. M.'s remarks on this subject. Too much attention can never be bestowed. Dr. T. also spoke of the frequent injury done to patient and operator by operating when not in a proper physical condition. Again, patients must be instructed, so that they may have a just appreciation of the value of their teeth. Many will give $50 or $100 for the restoring to soundness of an eye or an ear, when they would not give $5 for a tooth.

Next subject, "Absorption and reproduction of alveolar process, and its treatment."

Dr. Wilson—Absorption can be prevented, if timely treatment is given, the dentist having the ability to form a correct diagnosis of the case. The first, and all important item, is to impress the patient with the necessity of cleanliness. Is skeptical as to reproduction.

Dr. Morgan—Asked, if in the case of correcting irregularities, reproduction does not take place?

Dr. Wilson—Yes! but I do not consider that as coming directly under this head. Would make my remarks applicable only to those cases, where absorption was at the edge of the gum, causing the loosening of the teeth.

Dr. Nourse—Asked, if in correcting irregularities for patients, twenty-five years of age, there was a defect of the alveolar process, owing to non-reproduction?

Dr. Shadoan—Believes that the same reproduction would take place, as in the filling up of sockets after extraction.

Dr. Russell—Have corrected irregularities, and they now are sound and solid.

### Afternoon Session.

Subject, "Filling teeth, relative value of different material.

Dr. Nourse—Use adhesive foil principally, rarely amalgam. Use the burr, chisel and excavator in turn, if thin covering, over nerve introduce a drop of moist plaster of Paris as non-conductor. Gold in rope with broad point plugger, condensing thoroughly until full, then wedge when necessary with soft foil.

Dr. Russell—Uses gold, amalgam, and tin. Success depends largely on the ability of the operator.

Dr. Wilson—We operate to save teeth, and for this purpose gold only should be used; if other material is used the tooth is filled but not truly saved. Experiments with Wood's filling thinks that it will withstand action of acids of the mouth, but will permit the fluids to enter between the filling and the walls of cavity. I doubt the possibility of forcing it against the walls with sufficient firmness to exclude moisture, and have seen the teeth saturated in four weeks, and dentine turning yellow. For all *cheap* fillings, I prefer amalgam. If properly prepared it will better preserve a tooth, as it can be pressed against the walls. I use the mallet with great advantage in cutting away the enamel, also forming grooves for retention of gold in approximate cavities of central incisor. Use adhesive gold in pellets, commence at bottom of cavity pressing towards the walls, thereby filling its retaining points, instead of forcing or wedging into them from the mass. My instruments are squarely serrated instead of V shaped, for this idea I am indebted to Dr. Taft. I also use the coffer dam, which is very efficient in protecting the filling from moisture. Have been practicing building up the crowns of front teeth and cusps of molars, with success, and find the mallet a very important auxillary in this work.

Dr. Morgan—The unanimity of the experiences, as expressed by those here present, and with whom I agree, will require but few words to express my views. Amalgam being more dense will resist the wear of mastication better than Hill's stopping, Os Artificial, &c. I consider it next to gold, yet use it but seldom, except for childrens teeth. I use soft foil in blocks, it being more pliable can be manipulated with greater greater ease than any other kind; but as I have had no experience with adhesive foil, I will not dispute with its advocates.

Dr. Cobb—Each article has its advantage, each the plan peculiar to itself. I use soft foil for the body of filling and finish out with Abbey's No. 4 adhesive.

A resolution was here passed. That the physicians of Nashville be invited to participate at the discussion of Reproduction of Bone. Adjourned to 8 o'clock in the evening.

### Evening Session.

Subject, "Reproduction of Bone."

Dr. Taft—Has found that much benefit may be derived from the use of bone phosphate, in cases when the teeth do not appear to be properly developed. Considers it of great importance in cases where the parents have inferior teeth, that bone phosphate should be administered to mother during gestation and lactation, thereby furnishing the material through the mother, for the formation of a perfect set of teeth.

Dr. Garkey—Having travelled extensively in Europe, and having devoted much of his time to the thorough investigation of subjects interesting to the profession, gave a brief statement of the condition of the teeth in Germany. I visited a school of about 65 children, and was requested to examine their teeth. I found none decayed. Their ages varied from 6 to 12. This superior condition of their deciduous teeth is mainly attributable to the plain living and the full use of their masticatory organs. Rye bread, which contains the required material for the formation of bone, is the principle article of food, this also compels the proper use of them, to give them firmness in the sockets. Another remarkable fact, was, that the women all had bad teeth, this can only be accounted for, by the fact that they are not cleanly.

Dr. Taft—Suggests that the draught of bone material, during gestation, might account for this change, also that the fluids of the mouth undergo a change. In countries where their habits of life are more natural than in America, good teeth is the rule not the exception. The *Scotch*, from their use of rye bread, furnish the bone supply, also the required exercise; and this is as essential to the perfect development of the tooth, as of any organ of the body. An arm, if held long in disuse, will soon shrink, loose its strength, and become useless; disuse will effect decay.

Dr. Garkey—Many times the permanent teeth do not have sufficient room, when the deciduous teeth have vacated, owing to the non-enlargement of the arch; exercise is essential to the proper formation of the arch. Without this, irregularity is the result. It seems as though nature had committed an error, by giving teeth to children of the present age; as they are fed on such soft pulpy food, that only the gums are necessary to its mastication.

On motion, the subject of "Filling teeth" was resumed.

Dr. Garkey—I treat my patient according to the appreciation manifested. Dr. G. here gave the following experiment in treating nerve, and requested that all present should examine its merits, by experiments, and report at next meeting. Second sup molar posterior approximal surface, near the neck of tooth, nerve exposed, applied pure arsenic. Cut away the crown of tooth, and filled the original orifice, afterward the artificial cavity. I only destroy the nerve pulp, leaving the arsenic from one to four hours. After removal of pulp, apply cotton saturated with collodion, and let it remain from three days to a week; and have found after three weeks, that the vitality of the nerve in the roots was not impaired.

Dr. Morgan—Never leaves arsenic in tooth longer than four hours, and then finds the whole nerve in crown and fangs dead, in a large majority of cases.

Dr. Shadoan—Approves of Dr. Garkey's procedure in this class of operations. He had a case where the posterior lingnal surface at the margin of the gum of a second left superior molar. The nerve was exposed and, of course, the thorough extirpation or removal was impracticable through the opening. But in order to make thorough work, he drilled a sufficiently large hole in the grinding surface to admit of a free use of nerve instruments. Destroyed the nerve, removed it, and made a thorough operation.

As to other material than gold, he usually prefers Wood's fusible alloy. He thinks it better than amalgams, tin or artificial bone. He objects to cheap materials.

Dr. Taft—Gave a case of excessive sensitiveness of dentine, attributable to vitiated oral secretion, which was overcome by the use of iodide of potassæ, and phosphates.

Adjourned.

THURSDAY, 9 A. M.

Subject, "The best method of obtaining impressions."

Dr. Garkey—Request the patient to rinse the mouth with cold water, before the impression is removed, this causes it to be set firmly. I use wax for all patients except serious cases, and experience no trouble from expansion of wax. Dry the mouth with bibulous paper before introducing the wax.

Dr. Naghel—Gives this receipt for imp. wax: 1 lb. wax, 1 oz. burgundy pitch.

Dr. Wilson—For partial sets first uses wax, if not perfect fill impression with plaster and replace, always use wax for lower sets.

Dr. Bedford—Uses parefine and wax with success.

Dr. Fouche—Uses nothing but plaster, until recently, have been making use of wax first, but have not fully decided as to its merits.

Dr. Morgan—Asked for a remedy for the rocking of plates.

Dr. Garkey—I dip my rubber plate in hot parefine, then place in the mouth and press to position, the plate will retain its shape. To harden the model, first warm it, then dip into a solution of Borax. This will protect the model, also give better surface to the rubber plate.

Subject, "When and how should the different materials for plate be used."

Dr. Fouche—In a narrow arch, I use gold, a wide one rubber.

Dr. Wilson—For temporary sets, I insert plate in from three to six weeks after extraction; if on silver, use No. 37. For casts, take 8 parts bismuth, 5 lead. Pour the metal into a metal ring, ½ inch thick and 1½ inches wide, and dip my model having a hole through its centre for escape of air. Take cast thus obtained and coat with whiting, encircle with sheet-iron band, and pour warm metal. Always make duplicate dies. Oil dies, and swedge slightly, then thoroughly anneal before swedging fully, all the prominences, with first and best dies. Silver plates I solder in a continuous band. Rivet all teeth with a serrated pointed instrument. Give gold the preference for partial sets. Have nearly abandoned the soldering teeth on gold, find the combination of gold and rubber, using sectional teeth, much preferable. For solder, five dollar gold pieces, 1 dwt., 16 grs. silver, 20 grs. lead, 6 grs. copper.

"Neuralgia, what is it and how should it be treated."

Dr. Garkey—Is wholly in the dark as to its causes or its relief, more than by local warm applications.

